# Antimicrobial resistance of *Clostridioides difficile* in veterinary medicine around the world: A scoping review of minimum inhibitory concentrations

**DOI:** 10.1016/j.onehlt.2024.100860

**Published:** 2024-07-20

**Authors:** Mauricio Andino-Molina, Ines Dost, Mostafa Abdel-Glil, Mathias W. Pletz, Heinrich Neubauer, Christian Seyboldt

**Affiliations:** a Grupo de Investigación en Enfermedades de Etiología Microbiana (GIEEM) & Observatorio Universitario de Genómica y Resistencia Antimicrobiana (OUGRAM), Instituto de Investigaciones en Microbiología (IIM), Escuela de Microbiología, Facultad de Ciencias, Universidad Nacional Autónoma de Honduras, Honduras; b Institute of Bacterial Infections and Zoonoses, Friedrich-Loeffler-Institut, Jena, Germany; c Landesuntersuchungsamt Rheinland-Pfalz, Koblenz, Germany; d Institute for Infectious Diseases and Infection Control, Jena University Hospital, Jena, Germany

**Keywords:** *Clostridium difficile*, Clostridioides, Antimicrobial resistance, Veterinary, Animal, Antimicrobial susceptibility testing, One health

## Abstract

**Objective:**

To provide a comprehensive characterization of *Clostridioides difficile* antimicrobial resistance (AMR) data in veterinary medicine based on the minimum inhibitory concentrations (MICs) of all antimicrobial agents tested in relation to the techniques used.

**Methods:**

A systematic scoping review was conducted in accordance with the Preferred Reporting Items for Systematic Reviews and Meta-Analyses (PRISMA) extension for scoping reviews (PRISMA-ScR) and its associated checklist. The objective was to provide a synthesis of the evidence in a summarized and analyzed format.

To this end, three scientific databases were consulted: Scopus, PubMed, and Web of Science, up until December 2021. Subsequently, all identified literature was subjected to screening and classification in accordance with the established study criteria, with the objective of subsequent evaluation.

**Study selection and data extraction:**

A comprehensive analysis was conducted on studies regarding *Clostridioides difficile* antimicrobial resistance (AMR) in veterinary medicine across various animal species and related sources. The analysis included studies that presented data on antimicrobial susceptibility testing using the *E*-test, agar dilution, or broth microdilution techniques. The extracted data included minimum inhibitory concentration (MIC) values and a comprehensive characterization analysis.

**Results:**

A total of 1582 studies were identified in scientific databases, of which only 80 were subjected to analysis. The research on *Clostridioides difficile* antimicrobial resistance (AMR) in veterinary medicine is most prolific in Europe and North America. The majority of isolates originate from production animals (55%) and pets (15%), with pigs, horses, and cattle being the most commonly studied species. The tested agents' minimum inhibitory concentrations (MICs) and resulting putative antimicrobial resistance profiles exhibited considerable diversity across animal species and sources of isolation. Additionally, AMR characterization has been conducted at the gene and genomic level in animal strains. The *E*-test was the most frequently utilized method for antimicrobial susceptibility testing (AST). Furthermore, the breakpoints for interpreting the MICs were found to be highly heterogeneous and frequently observed regardless of the geographical origin of the publication.

**Conclusions:**

Antimicrobial susceptibility testing techniques and results were found to be diverse and heterogeneous. There is no evidence of an exclusive antimicrobial resistance pattern in any animal species. Despite the phenotypic and genomic data collected over the years, further interdisciplinary studies are necessary. Our findings underscore the necessity for international collaboration to establish uniform standards for *C. difficile* antimicrobial susceptibility testing (AST) methods and reporting. Such collaboration would facilitate a “One Health” approach to surveillance and control, which is of paramount importance.

## Highlights

• AMR data from 80 studies and >4600 isolates worldwide was assessed.
• AST methodologies and interpretative criteria are heterogeneous across literature.
• Production animals and pets' reports constitutes most of AMR data.
• AMR profiles are highly diverse across animal species.

## Graphical abstract

**Figure:**
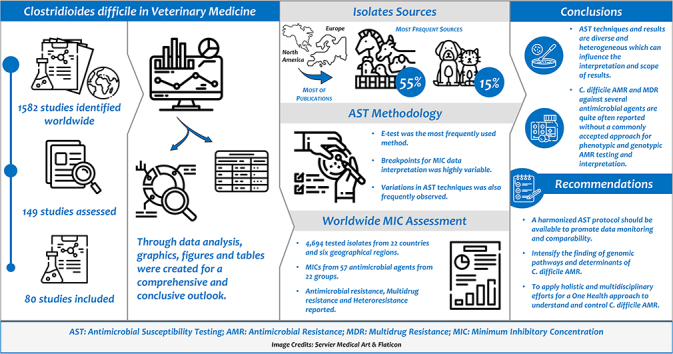

## 1 Introduction

*Clostridioides difficile* is a well-documented enteric anaerobic pathogen with a significant impact on hospital environments. Since its initial identification in 1935, the organism has been consistently documented globally [1,2]. *C. difficile* has been identified as the primary pathogen responsible for antibiotic-associated diarrhea, which results in a significant cumulative cost to healthcare systems [3]. The use of antimicrobial agents such as clindamycin, fluoroquinolones, and cephalosporins has been identified as a risk factor or inducer of *C. difficile* infection (CDI) in susceptible or vulnerable populations within hospital environments [3]*.*

The successful completion of antimicrobial susceptibility testing (AST) and laboratory diagnosis of *C. difficile* requires the presence of highly competent staff and the establishment of a robust infrastructure [4,5]. The consistent harmonization of technology and reporting style regarding minimum inhibitory concentration (MIC) data for antimicrobial agents remains a challenge in the effort for laboratory results comparability; standardized documentation for *C. difficile* AST and data interpretation is currently only available for human source isolates. Nevertheless, there is no consensus regarding clinical breakpoints for the majority of available antimicrobial agents [6].

The phenomenon of antimicrobial resistance (AMR) in strains of clinical origin in humans has been extensively studied [7]. These profiles are characterized by reports of high resistance to various classes of agents in the studied populations [8,9]. In a similar vein, although to a lesser extent, research into *C. difficile* AMR profiling has also been conducted in veterinary medicine [10]. Although, the known reports in veterinary medicine are of an isolated nature [11]. Consequently, it is necessary the integration and contextualization of this AMR data.

In recent years, there has been a growing interest in genomic assessment of strains of clinical and community origin due to the consistent and ongoing host overlapping presence of virulent *C. difficile* genotypes such as ribotype (RT) RT027, RT014, and RT078 [12]. The epidemiology of *C. difficile* infection (CDI) exhibits zoonotic components; while numerous reports on antimicrobial resistance have been published, it is evident that a comprehensive approach is required from the One Health perspective. This approach should permit the evaluation of antimicrobial resistance and its interrelated determinants in the environment, as well as in animal sources and their relationship with public health [13].

The objective of this study is to present a systematic collection of animal source data that will assist in contextualizing the magnitude of antimicrobial resistance in *C. difficile*. The integration of this data with evidence derived from environmental and clinical settings will enable the generation of scientific research that is based on agreed-upon methodologies and comparable results. These are essential for the advancement of this research field.

## 2 Methods

### 2.1 Protocol and registration

A scoping review was conducted in accordance with the Preferred Reporting Items for Systematic Reviews and Meta-Analyses Scoping Review Extension (PRISMA-ScR) and its checklist. The protocol stated that the aim was to investigate the patterns and profiles of *C. difficile* antimicrobial resistance (AMR) by means of minimal inhibitory concentration (MIC) assessment. The resulting data will assist us in understanding how AMR patterns are reported and distributed across the available literature. In this regard, the final protocol was registered at Open Science Framework on June 11, 2021 (https://osf.io/jcbs4/).

### 2.2 Search strategy and selection criteria

The search strategy was developed and refined in accordance with the population-exposure-outcome (PEO) search methodology approach, with input from our librarian expert. The consulted databases were PubMed (https://pubmed.ncbi.nlm.nih.gov/), Scopus (Elsevier, https://www.scopus.com/) and Web of Science (Clarivate Analytics, https://www.webofscience.com/wos/woscc/advanced-search) for the purpose of retrieving pertinent published literature until December 2021 in the English language. The search strategy included cross-sectional research, short communication studies, and review articles in which the abstracts described or reported *C. difficile* from animal or animal-related sources. Additionally, the search included articles that presented AST data using agar dilution, broth dilution, or *E*-test techniques. The retrieved data was meticulously evaluated to identify only those articles that presented their findings based on a proportion or number of tested samples. A more comprehensive and detailed list of inclusion/exclusion criteria, data charting, and our search strategy is available via our registered protocol and supplemental materials.

### 2.3 Data extraction

A revised, standardized tool was developed through a consensus process, and pertinent data and key findings were extracted from the screened literature. Special attention was paid to AMR data, with particular consideration given to the technique and MICs reported. In the event that the proportion or number of samples for a given minimum inhibitory concentration (MIC) category was absent from the data set, it was calculated from the available information. In instances where MIC ranges or data were derived also from human sources, particular attention was paid to MIC_90_ values for animal data.

### 2.4 Patient and public involvement

In this review project, no animals or human patients were involved.

## 3 Results

**Fig. 1:**
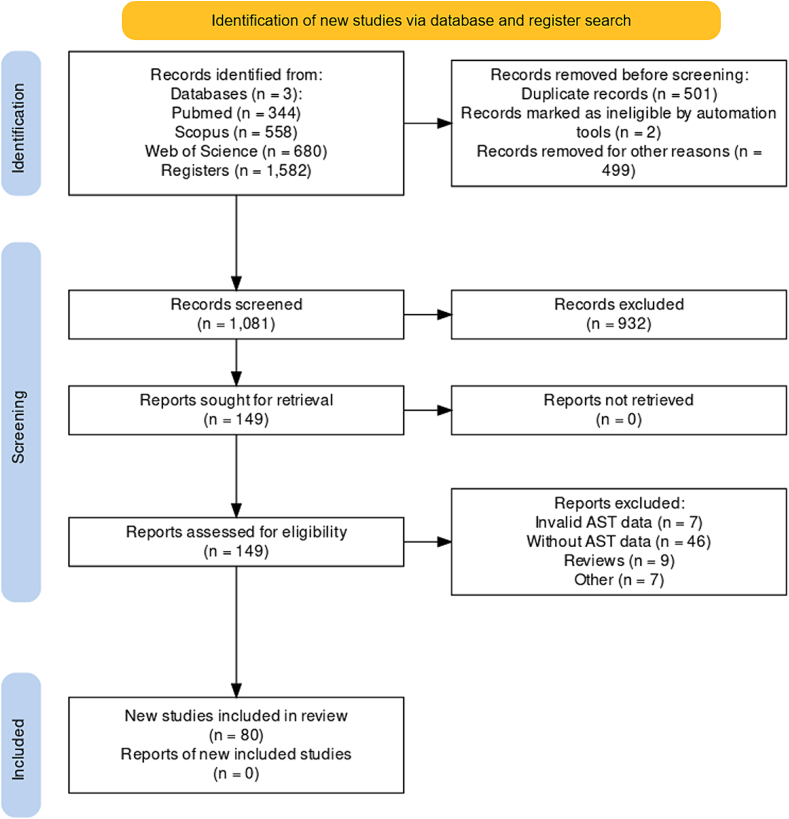
**Flow diagram for data screening and selection according to PRISMA-ScR (2018) and PRISMA (2020).** This flow diagram was created using the latest version available of Shiny app [102], available at: https://estech.shinyapps.io/prisma_flowdiagram/.

A search across three databases (PubMed, Scopus, and Web of Science) yielded 1582 studies. A total of 501 duplicates were identified and removed. After applying the inclusion criteria, 80 studies were subjected to analysis and data extraction (Fig. 1). A comprehensive list of key findings is presented in Table 1. The objective of all included studies was to characterize *C. difficile* antimicrobial resistance patterns.

**Table 1:** **Characterization of studies assessing *Clostridioides difficile* antimicrobial resistance in veterinary medicine (1997–2021).** Along with the listing, a description of the sources of isolation along with number of tested isolates and key findings are provided; the resistance or susceptibility data here presented correspond to criteria used by the authors and should be considered along with the epidemiological context. *A more detailed and extensive information is available in Supplemental Material Tables S1 and S2*.

Study No.	Citation in text	Publication's first author and year	Study type^1^	Region^2^	Country	Isolation source category	Sample description	Total of tested isolates	AST method	AST profiles and AMR reports^3^
1.	[14]	Jang, SS (1997)	Cross Sectional	North America	United States of America (the)	Production Animals	Diarrheical horses from 1 day to 27 years age	105	*E*-test and Agar Dilution	CHL, **RIF**, **VAN**, **MET**, **BAC**
2.	[15]	Båverud, V (1998)	Cross Sectional	Europe	Sweden	Production Animals	Mares (with/without acute colitis) and foals	9	NR	**RIF**, **ERY**
3.	[17]	Magdesian, KG (2002)	Cross Sectional Retrospective	North America	United States of America (the)	Production Animals	Foals (with diarrhea)	23	*E*-test	VAN, **MET**
4.	[16]	Marks, SL (2003)	Cross Sectional	North America	United States of America (the)	Pets	Dogs (diarrheic/ non-diarrheic)	70	Agar Dilution	VAN, MET
5.	[18]	Båverud, V (2003)	Cross Sectional	Europe	Sweden	Production Animals	Horses and foals, environment	52	*E*-test	CHL, **RIF**, VAN, **ERY**, MET, AMP, BEN, **BAC**, FUS, **TRS**
6.	[19]	Post, KW (2004)	Cross Sectional	North America	United States of America (the)	Production Animals	Neonatal pigs with enteritis	80	Agar Dilution	CTF, ERY, TIM, TYL, BAC, TET, VIR, TIA
7.	[20]	Magdesian, KG (2006)	Cross Sectional	North America	United States of America (the)	Production Animals	Horses with acute enteric disease, loose stools	130	*E*-test	VAN, **MET**
8.	[21]	Rodriguez-Palacios, A (2006)	Cross Sectional	North America	Canada	Production Animals	Calves (< 1 month, diarrheic/non-diarrheic)	30	*E*-test	VAN, **CLI**, MET, **LEV**
9.	[22]	Jhung, MA (2008)	Cross Sectional	North America	United States of America (the)	Production Animals	Bovine (8 mostly diahrreic calves), porcine (8 neonatal pigs with CDAD)	16	*E*-test	**CLI**, GAT, **LEV**, MOX
10.	[23]	Indra, A (2009)	Cross Sectional	Europe	Austria	Production Animals	Cow, pigs, and chickens	8	E-test	VAN, CLI, MET, MOX
11.	[24]	Norman, KN (2009)	Cross Sectional	North America	United States of America (the)	Production Animals	Pigs (all ages) and pre-lagoon effluent	131	*E*-test	AMC, **CHL**, **IMI**, **CXI**, VAN, **CLI**, MET, **AMP**, PIT, **CIP**, **TET**
12.	[25]	Debast, SB (2009)	Cross Sectional	Europe	Netherlands (the)	Production Animals	Neonatal pigs with diarrhea	5	*E*-test	VAN, CLI, ERY, MET, BEN, **CIP**, MOX
13.	[26]	Bakker, D (2010)	Cross Sectional	Europe	Netherlands (the)	Production Animals	Neonatal pigs with/without diarrhea	56	NR^5^	**TET**
14.	[27]	Thakur, S (2010)	Longitudinal	North America	United States of America (the)	Production Animals	Sows, young pigs	215	*E*-test	VAN, **ERY**, MET, **AMP**, **CIP**, **TET**
15.	[28]	Costa, MC (2011)	Longitudinal	North America	Canada	Production Animals	Veal calves	140	E-test	**TET**
16.	[29]	Harvey, RB (2011)	Cross Sectional	North America	United States of America (the)	Production Animals	Broiler chickens (42 days) and poultry meat	14	*E*-test	AMC, CHL, **IMI**, **CXI**, VAN, **CLI**, MET, AMP, PIT, **CIP**, **TET**
17.	[30]	Rodriguez-Palacios, A (2011)	Cross Sectional	North America	United States of America (the)	Production Animals	Fed and mature cattle	14	*E*-test	VAN, TIG, **CLI**, MET, **LIN**, **MOX**
18.	[31]	Thakur, S (2011)	Cross Sectional	North America	United States of America (the)	Wildlife/Sealife	Feral pigs	7	*E*-test	VAN, MET, **CIP**, **LEV**, **TET**
19.	[32]	Susick, EK (2012)	Longitudinal	North America	United States of America (the)	Production Animals	Pigs and piglets (7–10 days, weeks 4, 7, 16, 26), sows, environment (farms & slaughter), carcass	508	*E*-test	VAN, **ERY**, MET, **AMP**, **CIP**, **TET**
20.	[33]	Zidaric, V (2012)	Longitudinal	Europe	Belgium	Production Animals	Veal calves (day 14, 18, 25, 32, 46, 194)	38	*E*-test	**ERY**, MET, AMO, **DOX**, **TET**
21.	[34]	Fry, PR (2012)	Longitudinal	North America	United States of America (the)	Production Animals	Pig and piglets (farrowing, nursery and finishing) and sows	222	E-test	VAN, **ERY**, MET, **AMP**, **CIP**, **TET**
22.	[35]	Janezic, S (2012)	Cross Sectional	Europe	Slovenia	Mixed Sources	Piglets, calves, horse, poultry, birds, cats, dogs (symptomatic and asymptomatic animals)	18	*E*-test	CLI, ERY, PIT, MOX, TET, RIF
23.	[36]	Sthitmatee, N (2013)	Cross Sectional	Asia	Thailand	Wildlife/Sealife	Healthy elephants	15	Modified Agar Dilution	VAN, MET
24.	[37]	Peláez, T (2013)	Cross Sectional Retrospective	Europe	Spain	Production Animals	Piglets (1–7 days, diarrheic and non-diarrheic)	144	*E*-test	RIF, **ERT**, MER, TEI, VAN, TIG, **CLI,** DAP, **ERY**, MET^**4**^, LIN, **CIP**, **MOX**
25.	[38]	Álvarez-Pérez, S (2013)	Longitudinal	Europe	Spain	Production Animals	Piglets (day 8, 15, 22, 29, 36, 43, 50, 57, non-diarrheic), corresponding sows	41	E-test	RIF, **ERT**, MER, TEI, VAN, TIG, CLI, DAP, ERY, MET^**4**^, LIN, **CIP**, **LEV**, MOX, **OFL**, DOX
26.	[39]	Pirš, T (2013)	Cross Sectional	Europe	Slovenia	Mixed Sources	Pigs, ruminants, dogs, horse, poultry and poultry environment (water, liter, soil); most of animals without signs or symptoms of disease, human	96	Broth Microdilution	**STR**, AMC, AMS, CHL, **RIF**, **IMI**, **MER**, CFT, **CXI**, VAN, **TIG**, **CLI**, **DAP**, **ERY**, MET, LIN, **AMP**, MLC, **OXA**, **BEN**, PIP, PIT, **CIP**, **LEV**, **MOX**, **TET**, **TRS, GEN,** NIT, **QUD**
27.	[40]	Silva, ROS (2013)	Cross Sectional	Latin America	Brazil	Production Animals	Foals (diarrheic and non-diarrheic)	7	Agar Dilution	FLO, VAN, **ERY**, **TYL**, MET, **BEN**, OXY
28.	[41]	Wetterwik, KJ (2013)	Cross Sectional	Europe	Sweden	Pets	Dogs (8 month – 12 years, healthy and diarrheic)	4	*E*-test	VAN, CLI, ERY, **MET**, MOX
29.	[42]	Keessen, EC (2013)	Cross Sectional	Europe	Netherlands (the)	Production Animals	Piglets (1–7 days, diarrheic and non-diarrheic)	50	E-test	**IMI**, CUR, **CLI**, **ERY**, AMO, **CIP**, **MOX**, TET
30.	[43]	Avbersek, J (2014)	Cross Sectional	Europe	Slovenia	Production Animals	Goats (non-diarrheic), sheep (non-diarrheic and diarrheic), 1 day – 4 month, > 1 year	10	Broth Microdilution, E-test *(additionally only for CLI and ERY resistant strains)*	**STR**, AMC, AMS, CHL, RIF, IMI, MER, CFT, **CXI**, VAN, TIG, **CLI**, DAP, **ERY**, NIT, MET, LIN, **AMP**, MLC, **OXA**, **BEN**, PIP, PIT, **CIP**, **LEV**, MOX, TET, TRS, **GEN,** QUD
31.	[44]	Silva, ROS (2014)	Cross Sectional	Latin America	Brazil	Wildlife/Sealife	Crab-eating and hoary fox, cougar, oncilla, ocelot, maned wolf, jaguarundi, margay or tree ocelot (diarrheic and non-diarrheic animals)	2	Agar Dilution	FLO, VAN, ERY, TYL, MET, BEN, **OXY**
32.	[45]	Álvarez-Pérez, S (2014)	Cross Sectional	Europe	Spain	Wildlife/Sealife	40 zoo animal species (38 mammals, 2 birds)	7	*E*-test	RIF, **ERT**, **MER**, TEI, VAN, TIG, CLI, DAP, CLA, ERY, **MET**, LIN, AMO, **CIP**, **ENR**, **LEV**, MOX,
33.	[46]	Rodriguez, C (2014)	Cross sectional	Europe	Belgium	Production Animals	Foals and horses (mostly non-diarrheic)	12	*E*- test	MET, MOX
34.	[47]	Norman, K (2014)	Cross Sectional	North America	United States of America (the)	Production Animals	Pigs including: farrowing barn, nursery, breeding swine, growing/finisher swine	252	*E*-test	AMC, **CHL**, **IMI**, **CXI**, VAN, **CLI, MET**, **AMP**, PIT, **CIP**, **TET**
35.	[48]	Varshney, J (2014)	Cross Sectional	North America	United States of America (the)	Animal Related Product or Source	Meat: Beef, Pork, Chicken and Turkey	31	E-test	VAN, **CLI**, MET, **MOX**
36.	[49]	Noren, T (2014)	Cross Sectional	Europe	Sweden	Production Animals	Neonatal piglets and sows	42	*E*-test	RIF, VAN, CLI, MET, AMO, MOX, TET, **TRS**
37.	[50]	Silva, ROS (2014)	Cross Sectional	Latin America	Brazil	Mixed Sources	Piglets (CDI, diarrheic and healthy), dogs (diarrheic and healthy), foals (CDI, diarrheic and healthy), calves (diarrheic), ocelot (CDI), maned wolf (diarrheic).	41	Agar Dilution	VAN, **ERY**, **TYL**, MET, **BEN**, **OXY**, FLO
38.	[51]	Knetsch, C (2014)	Cross Sectional	Europe	Netherlands (the)	Production Animals	Pigs (asymptomatic)	21	*E*-test	**TET**
39.	[52]	Usui, M (2014)	Cross Sectional	Asia	Japan	Production Animals	Neonatal piglets (<20 days) and slaughter pigs	100	Agar Dilution	**CTR**, VAN, **CLI**, **ERY**, MET, **CIP**
40.	[53]	Spigaglia, P (2015)	Cross Sectional	Europe	Italy	Mixed Sources	Dogs (diarrheic and non-diarrheic), pigs (all with gastrointestinal syndrome)	101	*E*-test	**RIF**, VAN, **CLI**, **ERY**, **MET**, **MOX**
41.	[54]	Troiano, T (2015)	Cross Sectional	Europe	Italy	Wildlife/Sealife	Edible marine bivalve mollusks	36	E-test and Agar Dilution	VAN, **CLI**, **ERY**, FDX, MET, **CIP**, **MOX**
42.	[55]	Drigo, I (2015)	Cross Sectional	Europe	Italy	Production Animals	Rabbits (mostly with enteric disorders)	38	*E*-test	**RIF**, VAN, **CLI**, **ERY**, **MET**, **MOX**
43.	[56]	Álvarez-Pérez, S (2015)	Longitudinal	Europe	Spain	Pets	Dogs (puppies 7–55 days, and adult mothers)	34	E-test	RIF, **ERT**, TEI, VAN, TIG, **CLI**, DAP, **CLA**, **ERY**, MET^**4**^, LIN, AMO, **LEV**, MOX
44.	[57]	Cho, A (2015)	Cross Sectional	Asia	Republic of Korea (the)	Production Animals	Slaughter pigs	2	E-test	**CXI**, VAN, **CLI**, **ERY**, MET, AMP, **CIP**, **MOX**, TET
45.	[58]	Usui, M (2016)	Cross Sectional	Asia	Japan	Pets	Dogs (4 months-16 years, non-diarrheic)	68	Agar Dilution	**CTR**, VAN, **CLI**, **ERY**, MET, **CIP**, **LEV**, **SFX**
46.	[59]	Moono, P (2016)	Cross Sectional & Cohort	Oceania	Australia	Production Animals	Piglets (1–10 days, diarrheic and non-diarrheic)	29	Agar Dilution	SPE, TOB, AMC, MER, CTR, VAN, CLI, ERY, MET, PIT, MOX, TET, TRS, GEN
47.	[60]	Thitaram, S (2016)	Cross Sectional	North America	United States of America (the)	Production Animals	Healthy non-diarrheic porcines, and dairy & beef cattle	376	*E*-test	**AMC**, **RIF**, VAN, **CLI**, **ERY**, **MET**, **LIN**, **AMP**, **LEV**
48.	[61]	Wu, Y (2016)	Cross Sectional	Asia	Taiwan	Production Animals	Piglets, nursery pigs and sows	107	*E*-test	VAN, MET, **MOX**
49.	[62]	Knight, D (2016)	Cross Sectional	Oceania	Australia	Production Animals	Neonatal piglet, neonatal calf, foals, and kangaroo	80	Agar Dilution	AMC, RXM, MER, CTR, VAN, CLI, **ERY**, FDX, MET, PIT, MOX, **TET**, TRS
50.	[63]	Andres-Lasheras, S (2017)	Cross Sectional	Europe	Spain	Animal Related Product or Source	Pigs (fecal pool from pen floor), rodents, bird environmental, pig feed, intestinal contents (rat, mouse, pigeon)	34	*E*-test	VAN, **CLI**, **ERY**, MET, **MOX**, **TET**
51.	[64]	Orden, C (2017)	Cross Sectional	Europe	Spain	Pets	Dogs with digestive disorders	16	*E*-test	RIF, TEI, VAN, TIG, **CLI**, DAP, **ERY**, **MET**, **LEV**, **MOX**
52.	[65]	Álvarez-Pérez, S (2017)	Longitudinal	Europe	Spain	Pets	Dogs and cats from veterinary clinics	18	*E*-test	AMC, RIF, **IMI**, TEI, VAN, TIG, **CLI**, **ERY**, MET, LIN, **BEN**, **LEV**, TET
53.	[66]	Wu, Y (2017)	Cross Sectional	Asia	Taiwan	Animal Related Product or Source	Sampling at different points during process, including retail markets	39	E-test	VAN, MET, **MOX**
54.	[67]	Álvarez-Pérez, S (2017)	Cross Sectional Retrospective	Europe	Spain	Mixed Sources	Pigs (intensive raising and wild), dogs, poultry, zebras, iberian ibex	46	E-test	RIF, **ERT**, VAN, TIG, **CLI**, **ERY**, **MET**^**4**^, LIN, **LEV**, **MOX**, **TET**
55.	[68]	Bandelj, P (2017)	Cross Sectional Retrospective	Europe	Slovenia	Mixed Sources	Dairy cattle, environmental and animal-environmental-related sources	159	Broth Microdilution	**RIF**, IMI, CTR, VAN, TIG, **CLI**, **DAP**, **ERY**, MET, LIN, AMO, OXA, LEV, MOX, **TET**, FUS, TRS
56.	[69]	Knight, D (2017)	Cross Sectional Retrospective	Oceania	Australia	Production Animals	Piglets (<14 days)	16	Agar Dilution	SPE, TOB, AMC, RXM, MER, **CTR**, VAN, **CLI**, **ERY**, FDX, MET, PIT, MOX, **TET**, TRS, GEN
57.	[70]	Krutova, M (2018)	Cross Sectional	Europe	Czechia	Production Animals	Sows and piglets (diarrheical)	57	*E*-test	TET, VAN, MET, **MOX**
58.	[71]	Hampikyan, H (2018)	Cross Sectional	Europe	Turkey	Production Animals	Beef and sheep carcasses	161	MICE	**AMC**, **IMI**, **CTA**, **VAN**, **CLI**, **MET**, **AMP**, **TET**
59.	[72]	Orden, C (2018)	Cross Sectional	Europe	Spain	Animal Related Product or Source	Sandboxes of children and dog usage	20	*E*-test	AMC, RIF, **IMI**, TEI, VAN, TIG, **CLI**, **ERY**, MET, LIN, **BEN**, **LEV**, MIN, TET
60.	[73]	Dharmasena, M (2018)	Cross Sectional	North America	United States of America (the)	Animal Related Product or Source	Manure from feedstocks, compost	58	Agar Dilution	VAN, TIG, **CLI**, MET, LIN, MOX
61.	[74]	Andres-Lasheras, S (2018)	Cross Sectional	Europe	Spain	Pets	Dogs	6	*E*-test	VAN, **CLI**, **ERY**, **MET**, **MOX**, TET
62.	[75]	Andino-Molina, M (2019)	Cross Sectional	Latin America	Costa Rica	Production Animals	Piglets (diarrheic and non-diarrheic 1–14 days)	10	*E*-test	AMC, CHL, RIF, **CTA**, VAN, **CLI**, MET, LIN, AMO, MER, **AMP**, **ENR**, **LEV**, **TET**
63.	[76]	Kecerova, Z (2019)	Cross Sectional	Europe	Czechia	Production Animals	Horses (hospitalized and non-hospitalized)	18	*E*-test	VAN, **CLI**, **ERY**, MET, AMO, **ENR**, MOX, **TET**
64.	[77]	Weese, J (2019)	Cross Sectional	North America	Canada	Wildlife/Sealife	Polar bear samples (captive and wild)	24	*E*-test	CHL, VAN, **CLI**, MET, **CIP**, TET
65.	[78]	Rainha, K (2019)	Cross Sectional	Latin America	Brazil	Pets	Dogs (diarrheic) and post-treatment dogs (with antimicrobials)	5	*E*-test	VAN, **CLI**, MET
66.	[79]	Agnoletti, F (2019)	Cross Sectional	Europe	Italy	Wildlife/Sealife	Shellfish (mussels and clams)	113	E-test	**RIF**, **VAN**, **CLI**, **ERY**, MET^**4**^, **MOX**
67.	[80]	Pantuzza Ramos, C (2019)	Cross Sectional Retrospective	Latin America	Brazil	Pets	Lizards, chelonians, and snakes	2	E-test	VAN, **CLI**, MET, TRS
68.	[81]	Wei, Y (2019)	Cross Sectional	Asia	China	Pets	Dogs and cats (non-diarrheic and apparently healthy)	3	Agar Dilution	CHL, **CXI**, **VAN**, **CLI**, MET, **AMP**, MOX, TET
69.	[82]	Zhang, LJ (2019)	Cross Sectional Retrospective	Asia	China	Production Animals	Pigs (piglets and sows), chicken and duck	44	Agar Dilution	AMC, **CHL**, **IMI**, MER, **CXI**, **CTA**, CTF, VAN, **CLI**, **ERY**, FDX, MET, **AMP**, **CIP**, **MOX**, **TET**, FOS
70.	[83]	Rivas, L (2019)	Cross Sectional	Oceania	New Zealand	Animal related product or source	Animal faeces, wastewater and foods	12	*E*-test and Agar Dilution	AMC, MER, CTR, VAN, CLI, CLA, MET, BEN, MOX
71.	[84]	Barbanti, F (2020)	Cross Sectional Retrospective	Europe	Italy	Mixed sources	Pigs, mollusks, dogs, rabbits, horses, cattle, hares, cats, sheep and reptiles	125	Agar Dilution	**RIF**, VAN, **CLI**, **ERY**, **MET**, **MOX**
72.	[85]	Silveira, R (2020)	Cross Sectional	Latin America	Brazil	Pets	Cats (diarrheic and non-diarrheic)	6	*E*-test	VAN, **CLI**, MET, **CIP**
73.	[86]	Thanissery, R (2020)	Cross Sectional Retrospective	North America	United States of America (the)	Mixed sources	Dogs, cats, horses, ovines	39	*E*-test	**CTA**, VAN, **CLI**, **MET**, **CIP**, **LEV**
74.	[87]	Zhang, WZ (2020)	Cross Sectional Retrospective	Asia	China	Production animals	Calfs, sheeps	55	*E*-test	**CHL**, RIF, **MER**, VAN, **CLI**, **ERY**, MET, **CIP**, LEV, MOX, TET
75.	[88]	Bjoersdorff, OG (2021)	Cross Sectional	Europe	Denmark	Pets	Dogs	11	*E*-test	RIF, VAN, **CLI**, **ERY**, MET, MOX, **TET**
76.	[89]	Blasi, F (2021)	Cross Sectional	Europe	Italy	Production animals	Dairy and beef cattle; samples were taken from boots of workers as animal-related source	17	*E*-test	AMC, RIF, **VAN**, **CLI**, **ERY**, MET, **LIN**, AMP, **MOX**
77.	[90]	Taha Attia, AE (2021)	Cross Sectional	Middle East	Saudi Arabia	Animal related product or source	Chicken meat	11	E-test	VAN, CLI, MET, **MOX**, TET
78.	[91]	Taha, AE (2021)	Cross Sectional	Middle East	Saudi Arabia	Animal related product or source	Meat from camels, beef, sheep and goat	15	*E*-test	VAN, CLI, MET, **MOX**, TET
79.	[92]	Heise (2021)	Cross Sectional Retrospective	Europe	Germany	Animal related product or source	Chicken meat	51	Agar Dilution	RIF, VAN, **CLI**, **ERY**, MET, MOX
80.	[93]	Diniz, AN (2021)	Cross Sectional Retrospective	Latin America	Brazil	Production animals	Foals and horses with CDI	6	E-test	VAN, **CLI**, MET, **MOX**

1. Study type was determined according: a) as described in publication by the author(s); b) inferred from the study sampling and handling design.

2. As stated in the map disclaimer subsection on the manuscript, geographic regions do not correspond with any geopolitical view from FLI or author(s).

3. Abbreviations for antimicrobial agents were taken (when available) or inferred from EUCAST as distributed in “Antimicrobial abbreviations v7 20,220,120”.

Amoxicillin (AMO); Amoxicillin/Clavulanic acid (AMC); Ampicillin (AMP); Ampicillin/Sulbactam (AMS); Bacitracin (BAC); Cefotaxime (CTA); Cefotetan (CFT); Cefoxitin (CXI); Ceftiofur (CTF); Ceftriaxone (CTR); Cefuroxime (CUR); Chloramphenicol (CHL); Ciprofloxacin (CIP); Clarithromycin (CLA); Clindamycin (CLI); Daptomycin (DAP); Doxycycline (DOX); Enrofloxacin (ENR); Ertapenem (ERT); Erythromycin (ERY); Fidaxomicin (FDX); Florfenicol (FLO); Fosfomycin (FOS); Fusidic Acid (FUS); Gatifloxacin (GAT); Gentamicin (GEN); Imipenem (IMI); Levofloxacin (LEV); Linezolid (LIN); Meropenem (MER); Metronidazole (MET); Mezlocillin (MLC); Minocycline (MIN); Moxifloxacin (MOX); Nitrofurantoin (NIT); Ofloxacin (OFL); Oxacillin (OXA); Oxytetracyclin (OXY); Quinupristin/Dalfopristin (QUD); Penicillin or Benzylpencillin (BEN); Piperacillin (PIP); Piperacillin/Tazobactam (PIT); Rifampicin (RIF); Rifaximin (RXM); Sitafloxacin (SFX); Spectinomycin (SPE); Streptomycin (STR); Teicoplanin (TEI); Tetracycline (TET); Tiamulin (TIA); Tigecycline (TIG); Tilmicosin (TIM); Tobramycin (TOB); Trimethoprim/Sulfamethoxazole (TRS); Tylosin (TYL); Vancomycin (VAN); Virginiamycin (VIR).

Antimicrobial agents in **BOLD** correspond to those that have been classified by the authors as “**resistant**” in antimicrobial susceptibility testing reports. On the other hand, many antimicrobials do not currently have breakpoints, but high MICs have occasionally been reported, so antimicrobials that are *not in bold* should not be considered susceptible.

4. Metronidazole heteroresistance.

5. Abbreviation NR: Not explicitly reported.

### 3.1 Clostridioides difficile AMR data distribution and characterization

The antimicrobial susceptibility testing of *C. difficile* in veterinary medicine commenced in 1997 with the assessment of isolates from horses [14]. Since that time, numerous studies have been conducted, including investigations of isolates from a variety of animal species and sources. The findings of Jang et al. (1997) and Båverud et al. (1998) [14,15], provided the first insights into the diagnosis, management, and control of *C. difficile* in clinical veterinary microbiology. Subsequently, in 2003, isolates from dogs were analyzed, and it was proposed that genomic assessment, in addition to a more comprehensive phenotypic characterization, would provide further information for antibiotic treatment [16].

From the outset of the *C. difficile* AMR characterization, it was evident that the establishment of breakpoints for the tested antimicrobial agents would necessitate a consensus to render the data clinically relevant [6,16]. Nevertheless, despite the extensive analysis of a diverse range of literature, this objective has not been successfully achieved. The comparison of results using the breakpoint interpretation criteria set by the authors became highly complicated due to the complexity and high heterogeneity of the criteria. Consequently, alternative methodologies, such as the characterization and assessment of minimum inhibitory concentrations (MICs), were employed to elucidate the prevalence of *C. difficile* antimicrobial resistance and its impact on global public health.

**Fig. 2:**
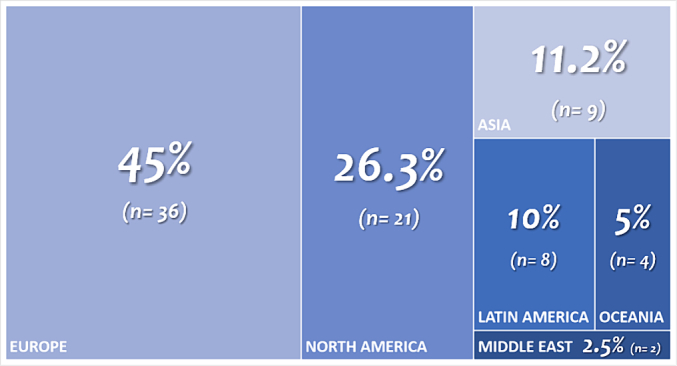
**Global distribution of studies assessing *Clostridioides difficile* in veterinary medicine.** After screening and data analysis, it was found that Europe was the highest literature contributor with 36 publications, followed by North America with 21 publications. Asia, Latin America, Oceania and Middle East contributed together with a total of 23 publications. Percentages have been adjusted by rounding.

A quantitative analysis of available data revealed that Europe and North America were responsible for the majority of research and knowledge contributions regarding *C. difficile* in veterinary medicine, collectively accounting for 45% and 26% of total published and indexed research, respectively (see Fig. 2). The first reports from Latin America and Asia did not emerge until 2013, followed by contributions from Oceania in 2014 and the Middle East in 2021 (Table 1).

**Fig. 3:**
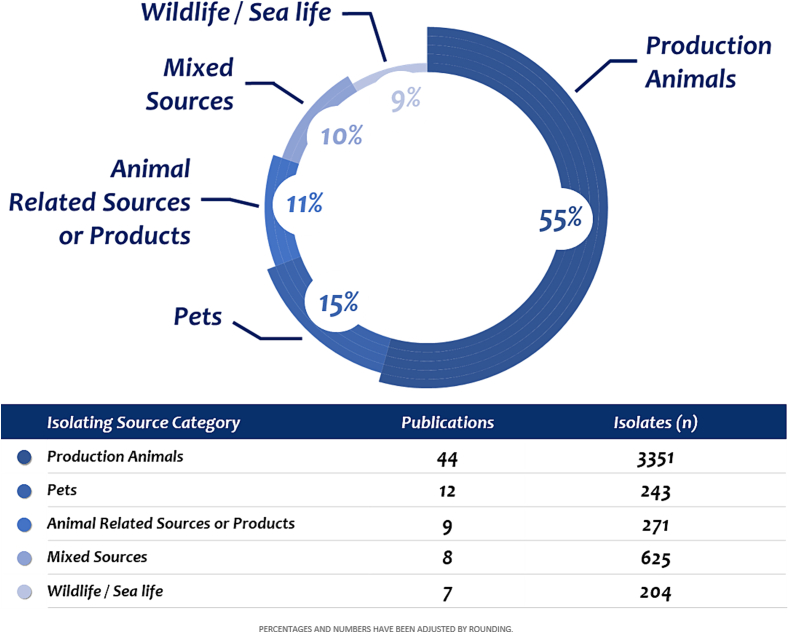
**Global characterization and distribution of *Clostridioides difficile* sources.** From analyzed literature that assesses antimicrobial resistance data in veterinary medicine, it was determined that most of samples from production animals came from pigs. Further information is available in Table 1 and S1.

From 4834 isolates, a total of 55% (2659) of the isolates tested originated from the bovine, avian, caprine, porcine, and equine production sector on a global scale. The most frequently analyzed production farm animals were pigs, followed by bovines and equines. Fifteen percent (725) of the strains were obtained from pets (dogs, cats, reptiles, and some birds), 11% (532) from animal-related products or sources (meat, environment, food products), 10% (483) from mixed sources (more than one category, or animal samples and environmental-related samples), and 9% (435) from wildlife and sea life, including zoo collections, captive animals, and mollusks (Fig. 3).

### 3.2 MICs distribution and patterns

**Fig. 4:**
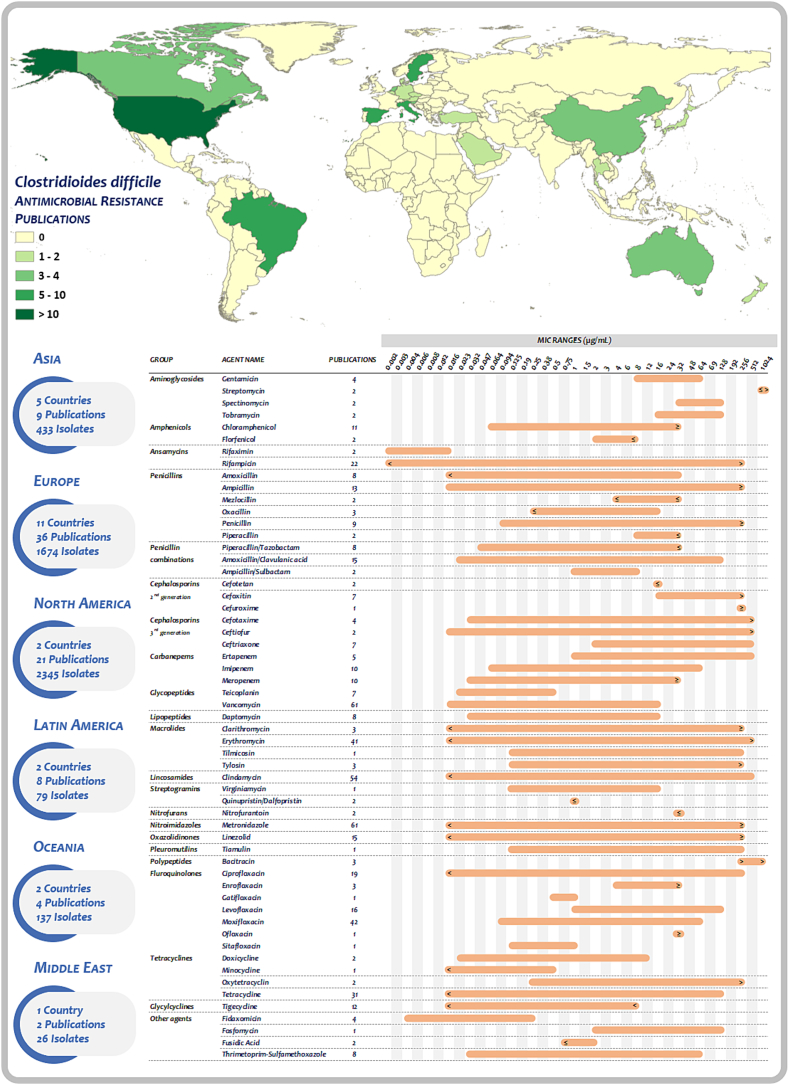
***Clostridioides difficile* worldwide publications and MIC ranges.** Antimicrobial susceptibility testing was performed in 4694 isolates from 22 countries against 57 antimicrobial agents or combinations of those. With some exceptions, MIC ranges correspond to the lowest and highest values reported across the analyzed regions. Detailed information regarding final MICs is available in Supplemental Materials (Table S1 and S2).

A total of 57 antimicrobial agents and 23 groups of agents were extracted from the literature for the purpose of determining their minimum inhibitory concentrations (MICs). A comprehensive and summarized overview of the minimum inhibitory concentrations (MICs) and ranges from six combined geographic regions is presented in Fig. 4. A detailed overview of MICs from individual regions is also available in Tables S1 and S2 of the supplementary materials.

#### 3.2.1 Aminoglycosides

Gentamicin was tested in four studies from Europe and Oceania. In Europe, the MICs of 106 isolates that came from production animals and mixed sources ranged between 8 and > 16 μg/ml [39,43]. Conversely, the MICs for 45 porcine isolates in Oceania were higher ranging between 16 and 64 μg/ml [59,69]. Streptomycin MICs were found to be in a range of ≤1000 – >1000 μg/ml in European isolates [39,43]. Regarding the MICs for spectinomycin and tobramycin, tested only in Oceania, concentrations reached up to 128 μg/ml for both agents [59,69].

#### 3.2.2 Amphenicols

In North America, 105 equine isolates demonstrated MICs for chloramphenicol between 1 and 8 μg/ml as previously reported [14]. In contrast, 512 isolates from farm pigs and wildlife (polar bears) had MICs between 0.5 and ≥ 32 μg/ml [24,47,77]. Notably, in ten porcine isolates from Latin America, MICs <32 μg/ml were found [75]. In Europe, the MICs for 106 isolates from mixed sources had MICs up to 16 μg/ml [39,43], while 52 equine and environmental isolates exhibited MICs up to 32 μg/ml [18]. For 47 Asian isolates, MICs ranged from 0.06 to 32 μg/ml [81,82]. In a study conducted in Brazil, the MIC for florfenicol was found to be up to 8 μg/ml [50]. Similarly, two isolates derived from wildlife exhibited MICs of ≤8 μg/ml [44].

#### 3.2.3 Ansamycins

Europe has the most extensive data set on the minimum inhibitory concentrations of rifampicin/rifampin. In some of the 353 isolates from production animals and mollusks, the MICs were >256 μg/ml [15,18,37,38,43,49,55,89]; in North America, the MICs from 481 production animal tested isolates were ≥ 128 μg/ml [14,60]. Notably, the strains with highest MIC values were found in equines [14]. In contrast, ten porcine isolates from Latin America exhibited MICs lower than 0.004 μg/ml [75]. In Oceania, 96 isolates with a similar origin to those previously described were tested against rifaximin. MICs ranged between 0.001 and 0.015 μg/ml [62,69].

#### 3.2.4 Penicillins

The most frequently tested penicillins were ampicillin and the combination piperacillin-tazobactam (Table S1). Ampicillin was tested in 1369 isolates from which MICs ≥256 μg/ml were reported in North American isolates [18,24,27,39,43,47,57,60,71,75,81,82,89]. Penicillin MICs ≥256 μg/ml were found in European isolates from animal-related sources [72] followed by isolates from European pets with MICs of ≥32 μg/ml in a total of 256 isolates tested [65]. In addition, amoxicillin was tested for 357 isolates (Table S1); MICs were up to 32 μg/ml in strains from Slovenian cattle and the environment [68]. Finally, only a limited number of reports on AST for mezlocillin (106 isolates) [39,43], oxacillin (265 isolates) [39,43,68] and piperacillin (106 isolates) [39,43] were available from Europe with MICs reports of ≤32 μg/ml in all sources and categories analyzed.

The combination of penicillins and β-lactamase inhibitors has been primarily tested in production animal strains. A total of 1272 isolates were tested for amoxicillin/clavulanic acid and their MICs ranged between 0.023 and 128 μg/ml (Table S1). The highest concentrations were observed in an Asian strain collection from ducks, pigs, and chickens [82]. In contrast, the MICs of 322 isolates, exclusively from Europe, exhibited a broader range spanning between ≤0.023 and ≥ 16 μg/ml [39,43,65,71,72,89]. MICs for piperacillin-tazobactam were found to be ≤32 μg/ml in 632 tested isolates [24,35,39,43,47,59,62,69]. Ampicillin/sulbactam was tested in 106 Slovenian isolates with MICs ranging between 1 and ≤ 8/4 μg/ml [39,43].

#### 3.2.5 Cephalosporins

High MICs of >256 μg/ml were observed for second-generation cephalosporin cefoxitin in three Asian strains from pets [81]. In a similar study, 46 strains from production animals yielded MICs between 16 and > 256 μg/ml [57,82]. A total of 106 European isolates from productive animals exhibited MICs >32 μg/ml [39,43]. Similarly, North America reported MICs of at least 64 μg/ml in some of its 383 strains tested [24,47]. The antimicrobial susceptibility testing against cefotetan showed MICs up to 16 μg/ml among Slovenian isolates from production animals [39,43]. Conversely, AST for cefuroxime in European porcine isolates yielded concentrations ≥256 μg/ml [42].

Of the third-generation cephalosporins, ceftriaxone was the most frequently tested agent, with a total of 464 isolates tested worldwide. MICs of ≥256 μg/ml were observed in isolates from Asia, while MICs up to 512 μg/ml were reported in Slovenian isolates in Europe [52,58,68]. A total of 137 isolates from Oceania exhibited MICs between 8 and 32 μg/ml for the same agent [59,62,69,83]. In Asia, MICs >512 μg/ml against cefotaxime and ceftiofur were detected among 44 strains from production animals [82]; in North America MICs against ceftiofur were > 256 μg/ml in 80 piglet strains [19]. In a lesser extent, MICs of ≥64 μg/ml were also detected for cefotaxime among 161 European isolates from production animals [71]; similarly, 49 isolates from North and Latin America showed MICs >32 μg/ml [75,86].

#### 3.2.6 Carbapenems

Meropenem was the most frequently tested carbapenem agent for *C. difficile* AST in veterinary medicine accounting for 478 isolates (Table S1). A total of 291 isolates from European production animals and related sources yielded MICs up to 4 μg/ml [[37], [38], [39],^43^]. Similar or even lower MICs were observed among 44 Asian isolates from similar sources, with values ranging between 0.03 and 2 μg/ml [82] and 137 isolates from Oceania exhibited MICs up to 2 μg/ml [59,62,69,83]. In the case of imipenem (941 tested isolates), 44 Asian strains yielded MICs between 0.06 and 64 μg/ml [82]; in Europe, MICs ≥32 μg/ml were reported within 514 isolates from diverse sources [39,42,43,65,68,71,72]. Additionally, MICs ≥16 μg/ml were also reported in 383 North American isolates [24,47]. Ertapenem was tested in 237 isolates from diverse sources in Europe with MICs ranging up to 512 μg/ml [37,38,67]. Strains from adult dogs and puppies were also tested against ertapenem in Spain with MICs ranging between 8 to ≥32 μg/ml [56]. Finally, a strain collection from a zoo in Spain yielded MICs ≥32 μg/ml for both, ertapenem and meropenem [45].

#### 3.2.7 Glycopeptides

The resulting worldwide data collection of 3726 isolates for vancomycin is more complex (Table S1). The highest MICs were reported in isolates from production animals from North America, yielding values up to 16 μg/ml [60]. MICs of 8 μg/ml or greater were observed in isolates from pets in Asia and Latin America [58,78,81]. Teicoplanin was tested in 279 European isolates from different source categories showing MICs between 0.023 and 0.5 μg/ml [37,38,45,56,64,65,72].

#### 3.2.8 Lipopeptides

Daptomycin MICs ranged from 0.032 to 16 μg/ml within 506 European isolates (Table S1). From those, strains isolated from pets exhibited MICs between 0.032 and 4 μg/ml [56,64]; Slovenian isolates from goats, sheep and lambs yielded MICs <4 μg/ml [43]. In Spain, the MIC values  for 185 pig isolates were up to 4 μg/ml [37,38]. Finally, the MICs of isolates from a Spanish zoo collection ranged between 0.25 and 1.5 μg/ml [45].

#### 3.2.9 Macrolides

Among the macrolides, erythromycin was the most frequently tested antimicrobial agent worldwide with 2369 isolates (Table S1). In Asia and Europe, MICs of erythromycin in production animal strains have been reported to exceed 512 μg/ml [37,68,82]. In the present study, all regions, with the exception of the Middle East, have reported MICs ≥256 μg/ml for the same agent in at least one of the analyzed categories. These findings are consistent with previous reports from other regions, including Europe, Oceania, and Asia [15,18,19,27,33,50,[52], [53], [54], [55], [56], [57], [58],^60^,^62^,^64^,^65^,^67^,^69^,^72^,^76^,^79^,^84^,^88^,^89^]. In European pet and production animal isolates, MICs of clarithromycin have been described as ≥256 μg/ml [56]; However, in Oceania, clarithromycin MICs were found to be between 0.38 and 4 μg/ml [83].

Tilmicosin and tylosin, which are only utilized in veterinary medicine, yielded MICs >256 μg/ml (Table S1). In strains from North American production animals, tilmicosin MICs exhibited values between 0.125 and 256 μg/ml [19]. Moreover, the MICs for tylosin were slightly higher in Latin American isolates, with values exceeding 256 μg/ml [50]. MICs up to 128 μg/ml were reported in porcine isolates from North America [19].

#### 3.2.10 Lincosamides

Clindamycin was widely tested in 2802 isolates (see Table S1). MICs up to 512 μg/ml were reported in strains from Slovenian isolates [68] and from porcine strains in Spain [37]. Similarly, high MICs up to ≥256 μg/ml were detected in isolates from all analyzed sources, with the majority originating from Europe [[53], [54], [55], [56],^64^,^65^,^67^,^72^,^76^,^79^,^84^,^88^,^89^] and a smaller proportion from Asia [52,57,58,82], North and Latin America [21,22,30,60,78,86]. Oceania reported MICs between 0.12 and > 32 μg/ml in isolates from highly diverse sources [59,62,69,83]. In the Middle East MICs of up to 4 μg/ml were observed in 26 isolates derived from meat of various animal species [90,91].

#### 3.2.11 Streptogramins

The antimicrobial Virginiamycin demonstrated the ability to inhibit the growth of 80 porcine isolates from North America, with MICs reaching up to 16 μg/ml [19]. Furthermore, quinupristin/dalfopristin was evaluated in 106 Slovenian isolates, with MICs ≤1 μg/ml [39,43].

#### 3.2.12 Nitrofurans

The reports for nitrofurans AST originate from 106 Slovenian strains with MICs ≤32 μg/ml [39,43].

#### 3.2.13 Nitroimidazoles

The data on metronidazole AST from 3468 isolates is highly diverse. A high proportion of strains from almost all analyzed source categories in Europe and North America exhibited MICs ≥256 μg/ml (Table S1). It is of concern that MICs equal or >256 μg/ml have been described in strains isolated from European pets [41,53,64,74,84], production animals [53,67] and zoo animals [45]. Similarly, the same high MICs were observed in North American strains isolated from production animals [60]. In Latin America, all tested isolates and categories exhibited MICs of <32 μg/ml [50,75,78,80,85]. In the Middle East, MICs were observed to reach up to 16 μg/ml in strains isolated from meat products [90,91]. It is noteworthy that no MICs exceeding 1 μg/ml or 8 μg/ml were observed in strains from Oceania [59,62,69,83] and Asia [36,52,57,58,61,66,81,82], respectively.

#### 3.2.14 Oxazolidines

Linezolid was tested in 1049 isolates worldwide with MICs ≥256 μg/ml observed in strains from production animals in North America [60]. In contrast, European strains of the same origin exhibited MICs not higher than 48 μg/ml [[37], [38], [39],^43^,^89^]. A total of 52 pet strains exclusively from Europe were tested against linezolid, with MICs not exceeding 1 μg/ml [56,65]. Furthermore, strains collected from a zoo exhibited MICs not higher than 2 μg/ml [45].

#### 3.2.15 Polypeptides

Antimicrobial susceptibility testing was conducted on a total of 237 isolates against Bacitracin in North American horses (1997), pigs (2004), and European horses and their environment (2003). The results are presented in Table S1. The highest MICs were observed in North American equine isolates with values exceeding 1024 μg/ml [14]. The MICs of the porcine isolates were found to be >256 μg/ml [19].

#### 3.2.16 Fluoroquinolones

**Fig. 5:**
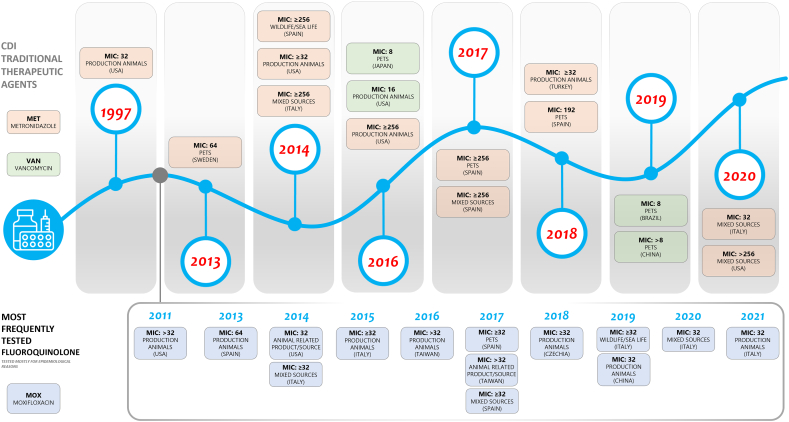
**Metronidazole, Vancomycin and Moxifloxacin High-MICs timeline.** As expected from a highly diverse collection of AST data, the MIC values for the selected agents are consistently variable. The sources of isolation for antimicrobial resistant strains include different animal species and the environment and show the wide-range of *C. difficile* AMR hosts and reservoirs. MICs are expressed in μg/ml. A complete description of MIC sources is available in Table S1.

Moxifloxacin was identified as the most frequently tested agent for 1765 isolates, as illustrated in Table S1. Fig. 5 presents a compilation of moxifloxacin reports from all analyzed categories with MICs ≥32 μg/ml [30,48,53,55,57,61,64,66,67,70,79,82,84,89]. The highest MICs were found in strains from Spanish piglets with values up to 64 μg/ml (Fig. 5) [37]. It is noteworthy that isolates from Oceania exhibited MICs up to 6 μg/ml [59,62,69,83]. For Ciprofloxacin, higher MICs with values up to 256 μg/ml were detected in Asian pets isolates [58], and MICs up to 64 and 128 μg/ml have been described for isolates of Asian and European production animals [37,52,82]. Similarly, high levofloxacin MICs were identified in Asian pet isolates with values reaching 128 μg/ml [58]. Finally, reports from North American for production animal and pet isolates yielded MICs of at least 32 μg/ml [21,22,60,86].

Enrofloxacin, tested in 34 isolates worldwide, showed MICs ≥32 μg/ml in strains isolated from European production [76] and wildlife animals [45]. In contrast, Latin American porcine isolates had MICs <8 μg/ml [75]. Ofloxacin MICs ≥32 μg/ml were found in 41 isolates from Spanish piglets [38]. In contrast, MICs up to 1 μg/ml for gatifloxacin have been described in 16 isolates from production animals in North America [22], and in 68 isolates from Asian dogs against sitafloxacin [58].

#### 3.2.17 Tetracyclines

Tetracycline was the most prevalent antimicrobial agent in this group, with MIC reports reaching 128 μg/ml in 124 isolates from production animals in Asia [82] and North America [19]. The MICs of European and Oceanian isolates were reported to be up to 32 μg/ml [67,69,76]. In 26 isolates of meat from different animal species from Saudi Arabia, MICs of 0.015 to 8.0 μg/ml have been reported [90,91]. The MICs for oxytetracycline were found to be >256 μg/ml in 43 isolates from Latin America [44,50]. Finally, MICs up to 12 μg/ml and 0.5 μg/ml were described for doxycycline [33] and minocycline [72], respectively.

#### 3.2.18 Glycylcyclines

The MICs of tigecycline in 68 European pet isolates ranged from 0.016 to 0.064 μg/ml [56,64]. The worldwide MICs for 209 production animal isolates ranged between 0.016 and 0.5 μg/ml [30,37,38,43], and up to 0.064 μg/ml in six strains from wildlife [45]. MICs up to 0.125 μg/ml were observed among Slovenian and Spanish isolates from mixed sources [39,67,68]. Twenty isolates from environmental sources and related to pets yielded MICs <0.016 μg/ml [72]. Finally, 58 environmental isolates from North America yielded MICs <8 μg/ml [73].

#### 3.2.19 Other antimicrobial agents

Fosfomycin MICs were determined for 44 strains of Asian productive animals, with values ranging from 2 to 128 μg/ml [82]. MICs of fusidic acid from 211 European isolates were ≤ 2.0 μg/ml [18,68]. Tiamulin MICs from 80 North American porcine isolates exhibited a range of 0.125 and 256 μg/ml [19]. The clinically relevant antimicrobial agent fidaxomicin, a macrocyclic agent, demonstrated MICs between 0.004 and 0.25 μg/ml in 96 Oceanian isolates analyzed [62,69]. In European isolates, the MICs were found to be <0.06 μg/ml [54]; production animal isolates from Asia demonstrated MICs up to 0.25 μg/ml [82]. Finally, trimethoprim/sulfamethoxazole was tested in 359 isolates from different sources in Europe with MICs ranging between 0.32/0.60 and > 32/608 μg/ml [18,39,43,49,68]. Furthermore, in 125 Oceanian isolates, the MICs were found to be higher, with values ranging from 16 to 64 μg/ml [59,62,69].

### 3.3 Antimicrobial agents commonly tested for assessing *C. difficile* AMR

A comparison of the AST results from the various geographic regions revealed that the most commonly tested agents across the analyzed literature were vancomycin, clindamycin, metronidazole, tetracycline, and moxifloxacin, with AST data entries from all regions. Furthermore, 83% (5/6) of the assessed regions had entries for erythromycin and amoxicillin/clavulanic acid. The considerable variability in the collection of AST data has demonstrated that there is no general consensus regarding the selection of antimicrobials for the assessment of AMR in *C. difficile* (Table S2).

As illustrated in Table 1, since the initial report of metronidazole-resistant *C. difficile* strains in equine isolates back in 1997 [14], reports of increased MICs up to >256 μg/ml have been published globally (Fig. 5). The strains exhibiting decreased susceptibility or resistance were isolated from a wide range of sources, including production animals and household pets [14,45,47,53,60,64,71,86]. Furthermore, to the complex timeline of high MICs for metronidazole, reports of increased MICs for vancomycin can be added, although these are quite scarce and originate from diverse sources, including pets [58,60,78,81]. In addition to these two CDI therapeutic agents, high MIC values and resistance for the fluoroquinolone moxifloxacin, which is frequently tested in *C. difficile* AST, have been reported [28,37,48,53,55,61,64,66,67,79,82,84,89].

### 3.4 Genomic characterization of *C. difficile* AMR across the analyzed literature in veterinary medicine

In some studies, included in this scoping review, not only phenotypic AMR were investigated but also genetic AMR determinants. As the determination of genetic AMR determinants did not influence the selection of publications included in this scoping review, this paragraph can just give a little insight in genetic AMR determinants of *C. difficile* in veterinary medicine.

#### 3.4.1 Tetracycline resistance

In some studies (Table S1), the assessment of AMR against tetracycline has been conducted by the detection of mobile genetic elements in conjuction with the phenotypic characterization of tetracycline MICs. In 2010, the genetic mobile element Tn*916*-like [26] was identified in all tetracycline-resistant porcine isolates. This was subsequently confirmed in 2013, when it was found in porcine isolates [42]. Moreover, additional strains, predominantly isolated from pigs, have demonstrated the presence of transposons Tn*6190* [51] and Tn*5397*-like [69]. Nevertheless, the detection of genetic mobile elements has been employed not only to trace the origin for antimicrobial resistance to tetracycline, but also to characterize it. For instance, *tet* gene variants have been identified in toxigenic and non-toxigenic strains from veal calves [28,33], swine [34,42,69], horses [76], dogs [88], and cattle [89].

#### 3.4.2 Fluoroquinolones and another agents' resistance

To a lesser extent, the resistance of fluoroquinolones has been assessed by investigating the gyrase genes across the literature. Mutations resulting in amino acid substitutions in GyrA from porcine isolates [42,61,70,82], and mutations resulting in amino acid substitutions in GyrB from equine [76], porcine [82], and avian isolates [82] have been identified. The presence of genetic resistance determinants, such as *erm* genes, were investigated to assess resistance to antimicrobial agents of the *macrolide-lincosamide-streptogramin B* (MLS_B_) group, which includes erythromycin and clindamycin. The gene *erm*(B) was found in strains from compost [73], horses [76] and dogs [78,88]. Furthermore, the genetic element *cfr* was identified in calves alongside *erm*(B) [89]. Finally, other AMR determinants including *lnuC*, *uppP2*, *terD1–4* or *cme*, and *blaR*, have also been fully described in strains isolated from pigs [69].

### 3.5 *C. difficile* AST methodology assessment

**Fig. 6:**
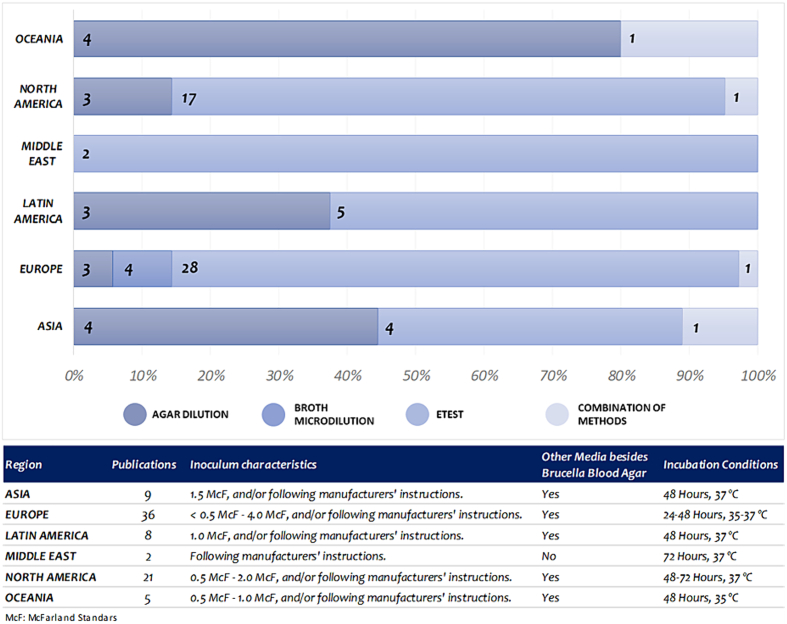
***Clostridioides difficile* antimicrobial susceptibility testing methodologies assessment.** Inoculum concentrations along with variations of media supplements were described across the analyzed literature. *E*-test was the most frequent AST method implemented.

With regard to laboratory methodology, 69.1% (57/80) of the published studies have employed the epsilometric gradient diffusion method known as *E*-test (E-TEST®, M.I.C.E™) as the methodology to assess *C. difficile* antimicrobial resistance in AST. Agar dilution methodology, which is considered the “*gold standard*”, was employed in 20.9% (17/81) of the studies, particularly in Oceania, Asia and Latin America. Only three studies from Slovenian researchers employed methods such as broth microdilution (Fig. 6).

The interpretative criteria and breakpoints selected were highly diverse across the analyzed data. To illustrate, despite the existence of CLSI [94] and EUCAST [95] breakpoints for metronidazole (CLSI: ≥32 μg/ml; EUCAST: >2 mg/L) and vancomycin (CLSI: No breakpoint available; EUCAST: >2 mg/L), the reported and used breakpoints for data interpretation were highly variable (Table S1). In general, modification or implementation of breakpoints derived from literature was observed as a common practice among researchers without a clear consensual rationale. Moreover, the source of interpretative criteria was frequently omitted despite the availability of standardized breakpoints provided by CLSI, EUCAST or the published literature (Table S1).

Finally, the selection and use of media, as well as the supplementation of blood-derived and specialized supplements such as vitamin K or hemin, varied according to the methodologies and incubation conditions reported. The most frequently reported incubation conditions were 24–48 h at 35–37 °C. Nevertheless, some publications omitted details regarding methodology, regardless of the region of origin (Table S1).

## 4 Discussion

After an exhaustive analysis of the literature, our objective was to provide a comprehensive characterization of antimicrobial resistance data from a clinical and categorical perspective. Indeed, after assessing the inherent complexity for comparability, we have characterized the antimicrobial resistance of *C. difficile* in veterinary medicine through a complete mapping of the isolate's sources and the evaluation of MICs reported against 57 antimicrobial agents (Fig. 4 and Supplemental Material). This provides a practical and consistent contextualization that, when considered alongside the global attention being paid to antimicrobial resistance reports, could contribute to methodological and interpretative harmonization of antimicrobial susceptibility testing and reporting.

*C. difficile* can be isolated from a wide variety of sources. The organism's presence in the environment [18,32,39,63,68], as well in pets, farm animals and exotic species such as domestic reptiles (Table 1) underscores its capacity for rapid dissemination and its potential to act as a zoonotic agent [13]. It is strongly advised that general antimicrobial resistance monitoring and surveillance in clinical settings be conducted from a “One Health” perspective [13]. However, it is crucial to exercise caution when interpreting AST results, considering the epidemiological and methodological context. Consequently, animals have been demonstrated to serve as potential reservoirs or amplifiers of AMR [96], yet the AMR profiles of *C. difficile* isolates from animals and humans may diverge from each other [87]. Consequently, the roles of the community [97], and the environment [73] in *C. difficile* epidemiology should be carefully considered.

*C. difficile* is also regarded as an AMR vector and amplifier due to its dynamic genomic asset of mobile elements [12,69,98]. It is of the utmost importance to comprehend these dynamics and to evaluate the genomic relationships between veterinary, human, and environmental strains, as well as the evolutionary processes that have shaped its contemporary epidemiology. It is possible that human and animal outbreak isolates with highly related AMR characteristics may have a common source of contamination, namely the acquisition of AMR genes. Indeed, independent genomic transfer of antimicrobial resistance genes from other Gram-positive bacteria, such as *Enterococcus* spp. or *Erysipelothrix rhusiopathiae* to *C. difficile* has been observed [99], in addition to transmission between humans and animals [51,69].

The occurrence of resistance to vancomycin and moxifloxacin, in addition to resistance and heteroresistance to metronidazole, has been documented in *C. difficile* strains over time, irrespective of host species and geographical region (Fig. 4). Furthermore, resistance to ciprofloxacin or enrofloxacin has been identified in animal isolates, as evidenced by data presented in Table 1. This suggests that selective pressure may be exerted in animal healthcare environments [76], or alternatively, that therapeutic administration may contribute to genetic mutations and quinolone resistance Consequently, it is imperative to conduct further investigation into this genetic evolution.

With regard to metronidazole, the results of the MIC assessments from our evaluated collection must be interpreted in light of the methodological variations resulting from the media dependency, heterogeneity, and instability of metronidazole resistance in *C. difficile* strains [100]. The various methodologies could potentially influence the results of antimicrobial resistance. It is evident that the implementation of standardized or harmonized methodological approaches for AST and MIC reporting assessment will significantly contribute to a more comprehensive understanding of the epidemiological dynamics underlying the obtained results.

## 5 Strengths and limitations

To the best of our knowledge, this review presents the first comprehensive collection of a large and complex dataset on antimicrobial resistance in *Clostridioides difficile* from animal or animal-related sources. It describes the minimum inhibitory concentrations instead of the clinical interpretation categories. This approach offers a more straightforward yet comprehensive overview of antimicrobial resistance. When available, laboratory techniques and genomic profiling were also included for assessment. The identification of potential avenues for further research endeavors, in addition to areas for improvement, was also outlined. Consequently, recommendations for a more comprehensive and harmonized reporting style were also presented.

In our view, a limiting factor that could contribute to the underestimation of high MICs and thus to the assessment of antimicrobial resistance is the inherent nature of the *E*-test (E-TEST™ or M.I.C.E.®) method. This method does not provide the final MIC of any commercially available antimicrobial agent when compared to the agar dilution method. Furthermore, the data in the analyzed collections may be incomplete or ambiguous. Moreover, although some studies present and analyze data exclusively from animal sources, some also include data from other related sources that undoubtedly contribute to the assessment of antimicrobial resistance in different settings. Nevertheless, the inclusion of all analyzed literature in this study is essential to highlight the research efforts made on this topic and to provide valuable insights for a more comprehensive understanding of *C. difficile* AMR in veterinary medicine.

## 6 Conclusions

In recent years, there has been a significant increase in the publication of detailed data on *C. difficile* antimicrobial resistance and its genetic determinants, particularly within the field of human medicine [12,98]. However and despite the existence of standardized techniques for AMR testing proposed by CLSI and EUCAST [94,95] or a simplified disk-diffusion technique proposed by the CA-SFM (*Committee of the Antibiogram of the French Society of Microbiology*) [101], the lack of a commonly accepted and used approach for both, phenotypic and genotypic AMR characterization is still noted. A standardized approach would facilitate the development of a comprehensive and globally comparable knowledge base on the ecology of *C. difficile* resistome.

The genetic diversity of *C. difficile* and its ability to infect a wide range of hosts observed in our analysis indicates a multifactorial epidemiology. The high degree of similarity observed between strains from animal and human hosts [51,69], provides a compelling rationale for research into a holistic epidemiological context. This is a perspective that has already been proposed in the current literature [13]. In light of the perplexing data regarding AMR and MIC patterns in veterinary medicine, which exhibit striking parallels to those observed in isolates from humans, it becomes evident that a “*One Health*” approach is imperative. This necessitates the formation of multidisciplinary teams to evaluate and validate practical approaches to combat *C. difficile* AMR infections in humans and animals. Such approaches should include optimal surveillance, testing, and control strategies, as well as the search for evidence-based therapeutic strategies that have a favorable impact on the health of patients and are in balance with animal health and a safe environment.

Consequently, it is now evident that there is an urgent need to harmonize AMR testing and to intensify the search for genomic pathways determining phenotypic behavior. In this context, it is imperative to develop a comprehensive protocol for future research that encompasses the following elements:

a) *The number and source of tested strains (a comprehensive metadata report)*
b) *Standard operating procedures for antimicrobial susceptibility testing*
c) *Minimum inhibitory concentrations, which could be reported individually or expressed in ranges along with concentration, and that should be grouped by metadata*
d) *Citation of interpretation criteria with a proper rationale*
e) *Proportion of resistant or susceptible strains for each tested antimicrobial agent*
f) *A cohesive report in respect to phenotype for genetic AMR elements*

There is an urgent need for greater collaborative efforts to establish a robust interdisciplinary research network that integrates insights from environmental, veterinary, and human medicine to accelerate the dissemination of research findings from the academic and research communities. This will ensure a long-term and sustainable environment for *C. difficile* research and control.

## Funding

MAM holds a research grant by DAAD (Research Grants – Doctoral Programmes in Germany, Code 57135739). ID is funded by European Union's Horizon 2020 Research and Innovation programme under grant agreement No 773830: One Health European Joint Programme.

## Map disclaimer

Maps used in our infographics, available thru repository access, were created for information and academic purposes and do not represent a geopolitical statement, from the authors and the correspondent affiliate institutions, regarding the aggrupation or representation of countries and territories. QGIS 3.24.3 Development team (2022). QGIS Geographic Information System. Open Source Geospatial Foundation Project. http://qgis.osgeo.org

## Patient consent for publication

N.A.

## Ethics approval

N.A.

## CRediT authorship contribution statement

**Mauricio Andino-Molina:** Writing – review & editing, Writing – original draft, Visualization, Validation, Resources, Project administration, Methodology, Investigation, Formal analysis, Data curation, Conceptualization. **Ines Dost:** Writing – review & editing, Visualization, Validation, Methodology, Formal analysis, Data curation. **Mostafa Abdel-Glil:** Writing – review & editing, Methodology, Data curation. **Mathias W. Pletz:** Writing – review & editing, Supervision, Methodology. **Heinrich Neubauer:** Writing – review & editing, Supervision, Methodology, Conceptualization. **Christian Seyboldt:** Writing – review & editing, Validation, Supervision, Resources, Project administration, Methodology, Conceptualization.

## Declaration of competing interest

N.A.

## Data Availability

Dataset containing all analyzed sources is available upon request to the authors and as supplementary material within the article.

## Appendix A Supplementary data

Supplementary material. Additional tables are provided here to allow for a more comprehensive interpretation and evaluation of the data. Image 1

## References

[1] Lawson P.A., Citron D.M., Tyrrell K.L., Finegold S.M. (2016). Reclassification of *Clostridium difficile* as Clostridioides difficile (Hall and O’Toole 1935) Prevot 1938. Anaerobe.

[2] Smits W.K., Lyras D., Lacy D.B., Wilcox M.H., Kuijper E.J. (2016). Clostridium difficile infection. Nat. Rev. Dis. Primers.

[3] Abad C.L.R., Safdar N. (2021). A review of Clostridioides difficile infection and antibiotic-associated diarrhea. Gastroenterol. Clin. N. Am..

[4] Somily A.M., Khan M.A., Morshed M. (2021). The laboratory diagnosis of Clostridioides difficile infection: an update of current laboratory practice. J. Infect. Dev. Ctries..

[5] Benkova M., Soukup O., Marek J. (2020). Antimicrobial susceptibility testing: currently used methods and devices and the near future in clinical practice. J. Appl. Microbiol..

[6] Sood A., Ray P., Angrup A. (2022). Antimicrobial susceptibility testing of anaerobic bacteria: in routine and research. Anaerobe.

[7] Saha S., Kapoor S., Tariq R., Schuetz A.N., Tosh P.K., Pardi D.S., Khanna S. (2019). Increasing antibiotic resistance in Clostridioides difficile: a systematic review and meta-analysis. Anaerobe.

[8] Spigaglia P., Mastrantonio P., Barbanti F. (2024). Antibiotic resistances of Clostridioides difficile. Adv. Exp. Med. Biol..

[9] Sholeh M., Krutova M., Forouzesh M., Mironov S., Sadeghifard N., Molaeipour L., Maleki A., Kouhsari E. (2020). Antimicrobial resistance in Clostridioides (Clostridium) difficile derived from humans: a systematic review and meta-analysis. Antimicrob. Resist. Infect. Control.

[10] Weese J.S. (2020). Clostridium (Clostridioides) difficile in animals. J. Vet. Diagn. Invest..

[11] Feldgarden M., Brover V., Gonzalez-Escalona N., Frye J.G., Haendiges J., Haft D.H., Hoffmann M., Pettengill J.B., Prasad A.B., Tillman G.E., Tyson G.H., Klimke W. (2021). AMRFinderPlus and the reference gene catalog facilitate examination of the genomic links among antimicrobial resistance, stress response, and virulence. Sci. Rep..

[12] O'Grady K., Knight D.R., Riley T.V. (2021). Antimicrobial resistance in Clostridioides difficile. Eur. J. Clin. Microbiol. Infect. Dis..

[13] Lim S.C., Knight D.R., Riley T.V. (2020). Clostridium difficile and one health. Clin. Microbiol. Infect..

[14] Jang S.S., Hansen L.M., Breher J.E., Riley D.A., Magdesian K.G., Madigan J.E., Tang Y.J., Silva J., Hirsh D.C. (1997). Antimicrobial susceptibilities of equine isolates of Clostridium difficile and molecular characterization of metronidazole-resistant strains. Clin. Infect. Dis..

[15] Baverud V., Franklin A., Gunnarsson A., Gustafsson A., Hellander-Edman A. (1998). Clostridium difficile associated with acute colitis in mares when their foals are treated with erythromycin and rifampicin for Rhodococcus equi pneumonia. Equine Vet. J..

[16] Marks S.L., Kather E.J. (2003). Antimicrobial susceptibilities of canine Clostridium difficile and Clostridium perfringens isolates to commonly utilized antimicrobial drugs. Vet. Microbiol..

[17] Magdesian K.G., Hirsh D.C., Jang S.S., Hansen L.M., Madigan J.E. (2002). Characterization of Clostridium difficile isolates from foals with diarrhea: 28 cases (1993-1997). J. Am. Vet. Med. Assoc..

[18] Baverud V., Gustafsson A., Franklin A., Aspan A., Gunnarsson A. (2003). Clostridium difficile: prevalence in horses and environment, and antimicrobial susceptibility. Equine Vet. J..

[19] Post K.W., Songer J.G. (2004). Antimicrobial susceptibility of Clostridium difficile isolated from neonatal pigs with enteritis. Anaerobe.

[20] Magdesian K.G., Dujowich M., Madigan J.E., Hansen L.M., Hirsh D.C., Jang S.S. (2006). Molecular characterization of Clostridium difficile isolates from horses in an intensive care unit and association of disease severity with strain type. J. Am. Vet. Med. Assoc..

[21] Rodriguez-Palacios A., Stampfli H.R., Duffield T., Peregrine A.S., Trotz-Williams L.A., Arroyo L.G., Brazier J.S., Weese J.S. (2006). Clostridium difficile PCR ribotypes in calves, Canada. Emerg. Infect. Dis..

[22] Jhung M.A., Thompson A.D., Killgore G.E., Zukowski W.E., Songer G., Warny M., Johnson S., Gerding D.N., McDonald L.C., Limbago B.M. (2008). Toxinotype V Clostridium difficile in humans and food animals. Emerg. Infect. Dis..

[23] Indra A., Lassnig H., Baliko N., Much P., Fiedler A., Huhulescu S., Allerberger F. (2009). Clostridium difficile: a new zoonotic agent?. Wien. Klin. Wochenschr..

[24] Norman K.N., Harvey R.B., Scott H.M., Hume M.E., Andrews K., Brawley A.D. (2009). Varied prevalence of Clostridium difficile in an integrated swine operation. Anaerobe.

[25] Debast S.B., van Leengoed L.A., Goorhuis A., Harmanus C., Kuijper E.J., Bergwerff A.A. (2009). Clostridium difficile PCR ribotype 078 toxinotype V found in diarrhoeal pigs identical to isolates from affected humans. Environ. Microbiol..

[26] Bakker D., Corver J., Harmanus C., Goorhuis A., Keessen E.C., Fawley W.N., Wilcox M.H., Kuijper E.J. (2010). Relatedness of human and animal Clostridium difficile PCR ribotype 078 isolates determined on the basis of multilocus variable-number tandem-repeat analysis and tetracycline resistance. J. Clin. Microbiol..

[27] Thakur S., Putnam M., Fry P.R., Abley M., Gebreyes W.A. (2010). Prevalence of antimicrobial resistance and association with toxin genes in Clostridium difficile in commercial swine. Am. J. Vet. Res..

[28] Costa M.C., Stampfli H.R., Arroyo L.G., Pearl D.L., Weese J.S. (2011). Epidemiology of Clostridium difficile on a veal farm: prevalence, molecular characterization and tetracycline resistance. Vet. Microbiol..

[29] Harvey R.B., Norman K.N., Andrews K., Hume M.E., Scanlan C.M., Callaway T.R., Anderson R.C., Nisbet D.J. (2011). Clostridium difficile in poultry and poultry meat. Foodborne Pathog. Dis..

[30] Rodriguez-Palacios A., Koohmaraie M., LeJeune J.T. (2011). Prevalence, enumeration, and antimicrobial agent resistance of Clostridium difficile in cattle at harvest in the United States. J. Food Prot..

[31] Thakur S., Sandfoss M., Kennedy-Stoskopf S., DePerno C.S. (2011). Detection of Clostridium difficile and Salmonella in feral swine population in North Carolina. J. Wildl. Dis..

[32] E.K. Susick, M. Putnam, D.M. Bermudez, S. Thakur, Longitudinal study comparing the dynamics of Clostridium difficile in conventional and antimicrobial free pigs at farm and slaughter, Vet. Microbiol. 157(1–2) (2012) 172–8. 10.1016/j.vetmic.2011.12.017 22243897

[33] Zidaric V., Pardon B., Dos Vultos T., Deprez P., Brouwer M.S., Roberts A.P., Henriques A.O., Rupnik M. (2012). Different antibiotic resistance and sporulation properties within multiclonal Clostridium difficile PCR ribotypes 078, 126, and 033 in a single calf farm. Appl. Environ. Microbiol..

[34] Fry P.R., Thakur S., Abley M., Gebreyes W.A. (2012). Antimicrobial resistance, toxinotype, and genotypic profiling of Clostridium difficile isolates of swine origin. J. Clin. Microbiol..

[35] Janezic S., Ocepek M., Zidaric V., Rupnik M. (2012). Clostridium difficile genotypes other than ribotype 078 that are prevalent among human, animal and environmental isolates. BMC Microbiol..

[36] Sthitmatee N., Warinrak T., Wongkalasin W. (2013). Susceptibility of isolated from healthy captive Asian elephants to metronidazole and vancomycin. Thai Journal of Veterinary Medicine.

[37] Pelaez T., Alcala L., Blanco J.L., Alvarez-Perez S., Marin M., Martin-Lopez A., Catalan P., Reigadas E., Garcia M.E., Bouza E. (2013). Characterization of swine isolates of Clostridium difficile in Spain: a potential source of epidemic multidrug resistant strains?. Anaerobe.

[38] Alvarez-Perez S., Blanco J.L., Pelaez T., Astorga R.J., Harmanus C., Kuijper E., Garcia M.E. (2013). High prevalence of the epidemic Clostridium difficile PCR ribotype 078 in Iberian free-range pigs. Res. Vet. Sci..

[39] Pirs T., Avbersek J., Zdovc I., Krt B., Andlovic A., Lejko-Zupanc T., Rupnik M., Ocepek M. (2013). Antimicrobial susceptibility of animal and human isolates of Clostridium difficile by broth microdilution. J. Med. Microbiol..

[40] Silva R.O., Ribeiro M.G., Palhares M.S., Borges A.S., Maranhao R.P., Silva M.X., Lucas T.M., Olivo G., Lobato F.C. (2013). Detection of a/B toxin and isolation of Clostridium difficile and Clostridium perfringens from foals. Equine Vet. J..

[41] Wetterwik K.J., Trowald-Wigh G., Fernstrom L.L., Krovacek K. (2013). Clostridium difficile in faeces from healthy dogs and dogs with diarrhea. Acta Vet. Scand..

[42] Keessen E.C., Hensgens M.P., Spigaglia P., Barbanti F., Sanders I.M., Kuijper E.J., Lipman L.J. (2013). Antimicrobial susceptibility profiles of human and piglet Clostridium difficile PCR-ribotype 078. Antimicrob. Resist. Infect. Control.

[43] Avbersek J., Pirs T., Pate M., Rupnik M., Ocepek M. (2014). Clostridium difficile in goats and sheep in Slovenia: characterisation of strains and evidence of age-related shedding. Anaerobe.

[44] Silva R.O., D’Elia M.L., Tostes Teixeira E.P., Pereira P.L., de Magalhaes Soares D.F., Cavalcanti A.R., Kocuvan A., Rupnik M., Santos A.L., Junior C.A., Lobato F.C. (2014). Clostridium difficile and Clostridium perfringens from wild carnivore species in Brazil. Anaerobe.

[45] Alvarez-Perez S., Blanco J.L., Martinez-Nevado E., Pelaez T., Harmanus C., Kuijper E., Garcia M.E. (2014). Shedding of Clostridium difficile PCR ribotype 078 by zoo animals, and report of an unstable metronidazole-resistant isolate from a zebra foal (Equus quagga burchellii). Vet. Microbiol..

[46] Rodriguez C., Taminiau B., Brevers B., Avesani V., Van Broeck J., Leroux A.A., Amory H., Delmee M., Daube G. (2014). Carriage and acquisition rates of Clostridium difficile in hospitalized horses, including molecular characterization, multilocus sequence typing and antimicrobial susceptibility of bacterial isolates. Vet. Microbiol..

[47] Norman K.N., Scott H.M., Harvey R.B., Norby B., Hume M.E. (2014). Comparison of antimicrobial susceptibility among Clostridium difficile isolated from an integrated human and swine population in Texas. Foodborne Pathog. Dis..

[48] Varshney J.B., Very K.J., Williams J.L., Hegarty J.P., Stewart D.B., Lumadue J., Venkitanarayanan K., Jayarao B.M. (2014). Characterization of Clostridium difficile isolates from human fecal samples and retail meat from Pennsylvania. Foodborne Pathog. Dis..

[49] Noren T., Johansson K., Unemo M. (2014). Clostridium difficile PCR ribotype 046 is common among neonatal pigs and humans in Sweden. Clin. Microbiol. Infect..

[50] Silva R.O.S., Oliveira Junior C.A., Diniz A.N., Alves G.G., Guedes R.M.C., Vilela E.G., Lobato F.C.F. (2014). Antimicrobial susceptibility of Clostridium difficile isolated from animals and humans in Brazil. Ciência Rural.

[51] Knetsch C.W., Connor T.R., Mutreja A., van Dorp S.M., Sanders I.M., Browne H.P., Harris D., Lipman L., Keessen E.C., Corver J., Kuijper E.J., Lawley T.D. (2014). Whole genome sequencing reveals potential spread of Clostridium difficile between humans and farm animals in the Netherlands, 2002 to 2011. Euro Surveill..

[52] Usui M., Nanbu Y., Oka K., Takahashi M., Inamatsu T., Asai T., Kamiya S., Tamura Y. (2014). Genetic relatedness between Japanese and European isolates of Clostridium difficile originating from piglets and their risk associated with human health. Front. Microbiol..

[53] Spigaglia P., Drigo I., Barbanti F., Mastrantonio P., Bano L., Bacchin C., Puiatti C., Tonon E., Berto G., Agnoletti F. (2015). Antibiotic resistance patterns and PCR-ribotyping of Clostridium difficile strains isolated from swine and dogs in Italy. Anaerobe.

[54] Troiano T., Harmanus C., Sanders I.M., Pasquale V., Dumontet S., Capuano F., Romano V., Kuijper E.J. (2015). Toxigenic Clostridium difficile PCR ribotypes in edible marine bivalve molluscs in Italy. Int. J. Food Microbiol..

[55] Drigo I., Mazzolini E., Bacchin C., Tonon E., Puiatti C., Bano L., Spigaglia P., Barbanti F., Agnoletti F. (2015). Molecular characterization and antimicrobial susceptibility of Clostridium difficile isolated from rabbits raised for meat production. Vet. Microbiol..

[56] Alvarez-Perez S., Blanco J.L., Pelaez T., Lanzarot M.P., Harmanus C., Kuijper E., Garcia M.E. (2015). Faecal shedding of antimicrobial-resistant Clostridium difficile strains by dogs. J. Small Anim. Pract..

[57] Cho A., Byun J.W., Kim J.W., Oh S.I., Lee M.H., Kim H.Y. (2015). Low prevalence of Clostridium difficile in slaughter pigs in Korea. J. Food Prot..

[58] Usui M., Suzuki K., Oka K., Miyamoto K., Takahashi M., Inamatsu T., Kamiya S., Tamura Y. (2016). Distribution and characterization of Clostridium difficile isolated from dogs in Japan. Anaerobe.

[59] Moono P., Putsathit P., Knight D.R., Squire M.M., Hampson D.J., Foster N.F., Riley T.V. (2016). Persistence of Clostridium difficile RT 237 infection in a Western Australian piggery. Anaerobe.

[60] Thitaram S.N., Frank J.F., Siragusa G.R., Bailey J.S., Dargatz D.A., Lombard J.E., Haley C.A., Lyon S.A., Fedorka-Cray P.J. (2016). Antimicrobial susceptibility of Clostridium difficile isolated from food animals on farms. Int. J. Food Microbiol..

[61] Wu Y.C., Lee J.J., Tsai B.Y., Liu Y.F., Chen C.M., Tien N., Tsai P.J., Chen T.H. (2016). Potentially hypervirulent Clostridium difficile PCR ribotype 078 lineage isolates in pigs and possible implications for humans in Taiwan. Int. J. Med. Microbiol..

[62] Knight D.R., Riley T.V. (2016). Clostridium difficile clade 5 in Australia: antimicrobial susceptibility profiling of PCR ribotypes of human and animal origin. J. Antimicrob. Chemother..

[63] Andres-Lasheras S., Bolea R., Mainar-Jaime R.C., Kuijper E., Sevilla E., Martin-Burriel I., Chirino-Trejo M. (2017). Presence of Clostridium difficile in pig faecal samples and wild animal species associated with pig farms. J. Appl. Microbiol..

[64] Orden C., Blanco J.L., Alvarez-Perez S., Garcia-Sancho M., Rodriguez-Franco F., Sainz A., Villaescusa A., Harmanus C., Kuijper E., Garcia M.E. (2017). Isolation of Clostridium difficile from dogs with digestive disorders, including stable metronidazole-resistant strains. Anaerobe.

[65] Alvarez-Perez S., Blanco J.L., Harmanus C., Kuijper E.J., Garcia M.E. (2017). Data from a survey of Clostridium perfringens and Clostridium difficile shedding by dogs and cats in the Madrid region (Spain), including phenotypic and genetic characteristics of recovered isolates. Data Brief.

[66] Wu Y.C., Chen C.M., Kuo C.J., Lee J.J., Chen P.C., Chang Y.C., Chen T.H. (2017). Prevalence and molecular characterization of Clostridium difficile isolates from a pig slaughterhouse, pork, and humans in Taiwan. Int. J. Food Microbiol..

[67] Alvarez-Perez S., Blanco J.L., Harmanus C., Kuijper E., Garcia M.E. (2017). Subtyping and antimicrobial susceptibility of Clostridium difficile PCR ribotype 078/126 isolates of human and animal origin. Vet. Microbiol..

[68] Bandelj P., Golob M., Ocepek M., Zdovc I., Vengust M. (2017). Antimicrobial susceptibility patterns of Clostridium difficile isolates from family dairy farms. Zoonoses Public Health.

[69] Knight D.R., Squire M.M., Collins D.A., Riley T.V. (2016). Genome analysis of Clostridium difficile PCR Ribotype 014 lineage in Australian pigs and humans reveals a diverse genetic repertoire and signatures of long-range interspecies transmission. Front. Microbiol..

[70] Krutova M., Zouharova M., Matejkova J., Tkadlec J., Krejci J., Faldyna M., Nyc O., Bernardy J. (2018). The emergence of Clostridium difficile PCR ribotype 078 in piglets in the Czech Republic clusters with Clostridium difficile PCR ribotype 078 isolates from Germany, Japan and Taiwan. Int J Med Microbiol.

[71] Hampikyan H., Bingol E.B., Muratoglu K., Akkaya E., Cetin O., Colak H. (2018). The prevalence of Clostridium difficile in cattle and sheep carcasses and the antibiotic susceptibility of isolates. Meat Sci..

[72] Orden C., Neila C., Blanco J.L., Alvarez-Perez S., Harmanus C., Kuijper E.J., Garcia M.E. (2018). Recreational sandboxes for children and dogs can be a source of epidemic ribotypes of Clostridium difficile. Zoonoses Public Health.

[73] Dharmasena M., Jiang X. (2018). Isolation of toxigenic Clostridium difficile from animal manure and composts being used as biological soil amendments. Appl. Environ. Microbiol..

[74] Andres-Lasheras S., Martin-Burriel I., Mainar-Jaime R.C., Morales M., Kuijper E., Blanco J.L., Chirino-Trejo M., Bolea R. (2018). Preliminary studies on isolates of Clostridium difficile from dogs and exotic pets. BMC Vet. Res..

[75] Andino-Molina M., Barquero-Calvo E., Seyboldt C., Schmoock G., Neubauer H., Tzoc E., Rodriguez C., Quesada-Gomez C. (2019). Multidrug-resistant Clostridium difficile ribotypes 078 and 014/5-FLI01 in piglets from Costa Rica. Anaerobe.

[76] Kecerova Z., Cizek A., Nyc O., Krutova M. (2019). Clostridium difficile isolates derived from Czech horses are resistant to enrofloxacin; cluster to clades 1 and 5 and ribotype 033 predominates. Anaerobe.

[77] Weese J.S., Salgado-Bierman F., Rupnik M., Smith D.A., van Coeverden de Groot P. (2019). Clostridium (Clostridioides) difficile shedding by polar bears (Ursus maritimus) in the Canadian Arctic. Anaerobe.

[78] Rainha K., Fernandes Ferreira R., Trindade C.N.R., Carneiro L.G., Penna B., Endres B.T., Begum K., Alam M.J., Garey K.W., Maria C.P. Domingues Regina, Ferreira E.O. (2019). Characterization of Clostridioides Difficile Ribotypes in Domestic Dogs in Rio de Janeiro, Brazil. Anaerobe.

[79] Agnoletti F., Arcangeli G., Barbanti F., Barco L., Brunetta R., Cocchi M., Conedera G., D'Este L., Drigo I., Spigaglia P., Mazzolini E. (2019). Survey, characterization and antimicrobial susceptibility of Clostridium difficile from marine bivalve shellfish of North Adriatic Sea. Int. J. Food Microbiol..

[80] Ramos C.P., Santana J.A., Morcatti Coura F., Xavier R.G.C., Leal C.A.G., Oliveira Junior C.A., Heinemann M.B., Lage A.P., Lobato F.C.F., Silva R.O.S. (2019). Identification and Characterization of *Escherichia coli*, Salmonella Spp., *Clostridium perfringens*, and *C. difficile* Isolates from Reptiles in Brazil. Biomed. Res. Int..

[81] Wei Y., Sun M., Zhang Y., Gao J., Kong F., Liu D., Yu H., Du J., Tang R. (2019). Prevalence, genotype and antimicrobial resistance of Clostridium difficile isolates from healthy pets in eastern China. BMC Infect. Dis..

[82] Zhang L.J., Yang L., Gu X.X., Chen P.X., Fu J.L., Jiang H.X. (2019). The first isolation of Clostridium difficile RT078/ST11 from pigs in China. PLoS One.

[83] Rivas L., Dupont P.Y., Gilpin B.J., Cornelius A.J. (2020). Isolation and characterization of Clostridium difficile from a small survey of wastewater, food and animals in New Zealand. Lett. Appl. Microbiol..

[84] Barbanti F., Spigaglia P. (2020). Microbiological characteristics of human and animal isolates of Clostridioides difficile in Italy: results of the Istituto Superiore di Sanita in the years 2006-2016. Anaerobe.

[85] Silva R.O.S., Ribeiro M.G., de Paula C.L., Pires I.H., Oliveira Junior C.A., Diniz A.N., de Araujo Nunes T.A., Lobato F.C.F. (2020). Isolation of Clostridium perfringens and Clostridioides difficile in diarrheic and nondiarrheic cats. Anaerobe.

[86] Thanissery R., McLaren M.R., Rivera A., Reed A.D., Betrapally N.S., Burdette T., Winston J.A., Jacob M., Callahan B.J., Theriot C.M. (2020). Clostridioides difficile carriage in animals and the associated changes in the host fecal microbiota. Anaerobe.

[87] Zhang W.Z., Li W.G., Liu Y.Q., Gu W.P., Zhang Q., Li H., Liu Z.J., Zhang X., Wu Y., Lu J.X. (2020). The molecular characters and antibiotic resistance of Clostridioides difficile from economic animals in China. BMC Microbiol..

[88] Bjoersdorff O.G., Lindberg S., Kiil K., Persson S., Guardabassi L., Damborg P. (2021). Dogs are carriers of Clostridioides difficile lineages associated with human community-acquired infections. Anaerobe.

[89] Blasi F., Lovito C., Albini E., Bano L., Dalmonte G., Drigo I., Maresca C., Massacci F.R., Orsini S., Primavilla S., Scoccia E., Tofani S., Forte C., Magistrali C.F. (2021). Clostridioides difficile in calves in Central Italy: prevalence, molecular typing, Antimicrobial Susceptibility and Association with Antibiotic Administration. Animals (Basel).

[90] Taha Attia A.E. (2021). Retail chicken meats as potential sources of Clostridioides difficile in Al-Jouf, Saudi Arabia. J Infect Dev Ctries.

[91] Taha A.E. (2021). Raw animal meats as potential sources of Clostridium difficile in Al-Jouf, Saudi Arabia. Food Sci Anim Resour.

[92] Heise J., Witt P., Maneck C., Wichmann-Schauer H., Maurischat S. (2021). Prevalence and phylogenetic relationship of Clostridioides difficile strains in fresh poultry meat samples processed in different cutting plants. Int. J. Food Microbiol..

[93] Diniz A.N., Cruz D.S.G., Ramos C.P., Oliveira C.A., Winter I.C., de Lima J.T.B., Carvalho A.D., Lobato F.C.F., Silva R.O.S. (2021). Clostridioides (Clostridium) difficile-associated diarrhea in equine in Minas Gerais, Brazil: clinical and microbiological characterization of six cases. Cienc. Rural.

[94] (2022). Clinical Laboratory Standards Institute, M100-S32: Performance Standards for Antimicrobial Susceptibility Testing.

[95] T.E.C.O.A.S (2023).

[96] Argudin M.A., Deplano A., Meghraoui A., Dodemont M., Heinrichs A., Denis O., Nonhoff C., Roisin S. (2017). Bacteria from animals as a Pool of antimicrobial resistance genes. Antibiotics (Basel).

[97] Fu Y., Luo Y., Grinspan A.M. (2021). Epidemiology of community-acquired and recurrent Clostridioides difficile infection. Ther. Adv. Gastroenterol..

[98] Spigaglia P. (2016). Recent advances in the understanding of antibiotic resistance in Clostridium difficile infection. Ther Adv Infect Dis.

[99] Kartalidis P., Skoulakis A., Tsilipounidaki K., Florou Z., Petinaki E., Fthenakis G.C. (2021). Clostridioides difficile as a dynamic vehicle for the dissemination of antimicrobial-resistance determinants: review and in silico analysis. Microorganisms.

[100] Boekhoud I.M., Hornung B.V.H., Sevilla E., Harmanus C., Bos-Sanders I., Terveer E.M., Bolea R., Corver J., Kuijper E.J., Smits W.K. (2020). Plasmid-mediated metronidazole resistance in Clostridioides difficile. Nat. Commun..

[101] Dubreuil L., C.-S (2020). Members of the, Improvement of a disk diffusion method for antibiotic susceptibility testing of anaerobic bacteria. French recommendations revisited for 2020. Anaerobe.

[102] Haddaway N.R., Page M.J., Pritchard C.C., McGuinness L.A. (2022). PRISMA2020: an R package and shiny app for producing PRISMA 2020-compliant flow diagrams, with interactivity for optimised digital transparency and open synthesis. Campbell Syst. Rev..

